# Interface traps and quantum size effects on the retention time in nanoscale memory devices

**DOI:** 10.1186/1556-276X-8-369

**Published:** 2013-08-29

**Authors:** Ling-Feng Mao

**Affiliations:** 1 Institute of Intelligent Structure and System, School of Urban Rail Transportation, Soochow University, Suzhou 215006, China

**Keywords:** Interface trap, Nanocrystalline, Quantum-size effect, Memory, Retention time, 85.30.Tv, 85.35.-p, 73.63.-b

## Abstract

Based on the analysis of Poisson equation, an analytical surface potential model including interface charge density for nanocrystalline (NC) germanium (Ge) memory devices with p-type silicon substrate has been proposed. Thus, the effects of P_b_ defects at Si(110)/SiO_2_, Si(111)/SiO_2_, and Si(100)/SiO_2_ interfaces on the retention time have been calculated after quantum size effects have been considered. The results show that the interface trap density has a large effect on the electric field across the tunneling oxide layer and leakage current. This letter demonstrates that the retention time firstly increases with the decrease in diameter of NC Ge and then rapidly decreases with the diameter when it is a few nanometers. This implies that the interface defects, its energy distribution, and the NC size should be seriously considered in the aim to improve the retention time from different technological processes. The experimental data reported in the literature support the theoretical expectation.

## Background

The performance and reliability of metal-oxide semiconductor is significantly influenced by the quality of the grown Si/SiO_2_ interface. The interface trap as a function of energy in the Si band gap exhibits two peaks, 0.25 and 0.85 eV for Si(110)/SiO_2_ interface [1] and 0.31 and 0.84 eV for Si(111)/SiO_2_ interface [2]. The P_b_ center resides on flat surfaces (terraces), not at ledges [3]; it is considered as the main source of defects at the Si(111)/SiO_2_ interface. It was named as P_b0_ with reference to the P_b1_ center on Si(100). The interface defect is amphoteric that is a donor level below mid gap and an acceptor level above mid gap. Memory structures based on nanocrystalline (NC) semiconductor have received much attention for next-generation nonvolatile memory devices due to their extended scalability and improved memory performance [4-6]. Recently, the quantum size effects caused by the channel material NC Si neglecting the interface charge on the threshold voltage of thin-film transistors without float gate [7] and on charging the dynamics of NC memory devices [8] have been studied. Here, both the quantum size effects caused by the float gate material NC and the interface traps effects on the retention time of memory devices are studied.

### Theory

For p-type silicon, Poisson's equation can be written as follows:

(1) $$\frac{\partial^{2}\psi }{\partial x^{2}}=-\frac{q\;}{\epsilon_{s}}\left(N_{A}\left(e^{-\frac{\mathrm{q\psi}}{\mathrm{kT}}}-1\right)-\frac{n_{i}^{2}}{N_{A}}\left(e^{\frac{\mathrm{q\psi}}{\mathrm{kT}}}-1\right)\right)\;$$

where *φ*(z) is the electrostatic potential, *ϵ*_s_ is the dielectric constant of silicon, *N*_A_ is the ionized acceptor concentrations, *n*_i_ is the intrinsic density, *k* is the Boltzmann constant, and *T* is the temperature. Using the relationship $\frac{\partial }{\partial z}{\left[\frac{\partial \varphi }{\partial z}\right]}^{2}=2\frac{\partial \varphi }{\partial z}\frac{\partial^{2}\varphi }{\partial z^{2}}$ and then integrating from 0 to *φ*_*s*_, obtain surface electric field at the side of silicon substrate as follows:

(2) $$E_{S}=\pm \sqrt{\frac{2\mathrm{qkT}N_{A}\;}{\epsilon_{s}}\left(e^{-\frac{q\psi_{S}}{\mathrm{kT}}}-1+\frac{q\psi_{S}}{\mathrm{kT}}+\frac{n_{i}^{2}}{{\left(N_{A}\right)}^{2}}\left(e^{\frac{q\psi_{S}}{\mathrm{kT}}}-1-\frac{q\psi_{S}}{\mathrm{kT}}\right)\right)\;}\;$$

If *ψ*_s_ > 0, choose the ‘+’ sign (for a p-type semiconductor), and if *ψ*_s_ < 0, choose the ‘−’ sign. Poisson's equation in the gate oxide and the NC Ge layer with uniformly stored charge density *Q*_nc_ per unit area can be written as follows:

(3) $$\frac{\partial^{2}\psi }{\partial x^{2}}=0\;$$

(4) $$\frac{\partial^{2}\psi }{\partial x^{2}}=-\frac{Q_{\mathrm{nc}}}{\epsilon_{\mathrm{nc}}d_{\mathrm{nc}}}\;$$

where *d*_nc_ and *ϵ*_nc_ are the thickness and the average dielectric constant of NC Ge layer, respectively. Consider boundary conditions for the case of interface charge density *Q*_it_ captured by the traps at Si/SiO_2_ interface; thus, the electric field across the tunneling oxide layer is the following:

(5) $$E_{\mathrm{ox}}=-\frac{-\epsilon_{S}E_{S}+Q_{\mathrm{it}}}{\epsilon_{\mathrm{ox}}}\;$$

where *ϵ*_ox_ is the dielectric constant of SiO_2_. The applied gate voltage of a NC flash memory device is equal to the sum of the voltage drop across the layer of NC Ge, SiO_2_, and p-Si:

(6) $$\begin{array}{ll} V_{g}= & -Q_{\mathrm{nc}}\left(\frac{d_{\mathrm{nc}}}{2\epsilon_{\mathrm{nc}}}+\frac{d_{\mathrm{cox}}}{\epsilon_{\mathrm{ox}}}\right)+\left(\epsilon_{S}E_{S}-Q_{\mathrm{it}}\right)\\ & \times \left(\frac{d_{\mathrm{tox}}}{\epsilon_{\mathrm{ox}}}+\frac{d_{\mathrm{nc}}\;}{\epsilon_{\mathrm{nc}}}+\frac{d_{\mathrm{cox}}}{\epsilon_{\mathrm{ox}}}\right)+\psi_{S}\; \end{array}$$

where *d*_tox_ and *d*_cox_ are the thickness of the tunneling oxide layer and control oxide layer, respectively. The interface charge density is obtained by multiplying the density of interface trap states (*D*_it_) by the trap occupation probability and integrating over the bandgap [9]:

(7) $$Q_{\mathrm{it}}=q\int D_{\mathrm{it}}\left(E\right)F\left(E\right)\mathrm{dE}$$

The Fermi-Dirac distribution function *F*(*E*) for donor interface traps is (1 + 2 exp[(*E*_*F*_ − *E*)/(*kT*)])^−1^ and that for the acceptor interface traps is (1 + 4 exp[(*E* − *E*_*F*_)/(*kT*)])^−1^.

The leakage current can be calculated using [10]:

(8) $$J=\int_{0}^{\infty }\frac{qm^{*}\mathrm{kT}}{2\pi^{2}\hbar^{3}}T\left(E\right)\ln\left(\frac{1+\exp\left(\left(E_{F}-E\right)/\mathrm{kT}\right)}{1+\exp\left(\left(E_{F}-E-\mathrm{qV}\right)/\mathrm{kT}\right)}\right)\mathrm{dE}$$

where *T*(*E*) is the transmission coefficient calculated by solving Equation 8 using the transfer matrix method, *V* is the voltage drop values in the tunneling region, m* is the effective electron mass, and *ħ* is the reduced Planck constant. The energy of the highest valence state (*E*_v_) and the energy of the lowest conduction state (*E*_c_) for spherical NCs of the diameter *d* (given in nanometer) are given by the following expression [4]:

(9) $$E_{c}\left(d\right)=E_{c}(\infty )+\frac{11863.7}{d^{2}+2.391d+4.252}\;\left(\mathrm{meV}\right)$$

(10) $$E_{v}\left(d\right)=E_{v}(\infty )-\frac{15143.8}{d^{2}+6.465d+2.546}\;\left(\mathrm{meV}\right)$$

The mean diameter (*d*) of NC Ge is uniquely controlled by the nominal thickness (*t*) of the deposited amorphous Ge using molecular beam epitaxy according to the law *d* ≅ *Kt* (K approximately 7), and the average density of NC Ge *D*_NC_ ≅ 6 × 10^*−3*^*/t*^2^[5]. Thus, the filling factor that is the ratio of area of NC Ge to total area can be obtained as 0.2349. The size-dependent dielectric constant can be obtained as follows [6]:

(11) $$\epsilon \left(d\right)=1+\left(\epsilon_{b}-1\right)/\left(1+{\left(2d_{0}/d\right)}^{1.1}\right)$$

where *ϵ*_b_ is dielectric constant of bulk Ge. The characteristic radius for Ge is 3.5 nm. Considering the fill factor, the average dielectric constant of NC Ge layer can be estimated using parallel capacitor treatment.

The top of the valence band of p-type silicon bends upward (*ψ*_s_ < 0 and *Ε*_s_ < 0) which causes an accumulation of majority carriers (holes) near the interface. Thus, the interface traps capture more holes when the float gate has been charged with electrons [9]. It results that the electric field across the tunneling oxide layer increases according to Equation 5, the transmission coefficient through the tunneling oxide layer increases, and the retention time decreases. Whereas, the top of the valence band of n-type silicon bends upward which causes a depletion of majority carriers (electrons) near the interface, and the interface traps capture less holes or capture electrons if the band bends even more so that the Fermi is level below mid gap [9]. Thus, it results that the electric field across the tunneling oxide layer decreases, the transmission coefficient decreases, and the retention time increases. Additionally, such a method is still valid for metal (or other semiconductor) NC memory in just using their equations to substitute Equations 9, 10, and 11 for NC Ge.

## Methods

The transfer matrix method used in the calculation of the transmission coefficient for the tunneling current can be described as the following. The transmission coefficient *T*(*E*_x_) was calculated by a numerical solution of the one-dimensional Schrödinger equation. A parabolic *E*(*k*) relation with an effective mass m* as parameter was assumed in the calculation. The barrier was discretized by *N* partial subbarriers of rectangular shape that covered the whole oxide layer of thickness. From the continuity of wave function and quantum current density at each boundary, the transmission coefficient is then found by:

(12) $$T\left(E\right)=\frac{m_{0}}{m_{N+1}}\frac{k_{N+1}}{k_{0}}\frac{\left|\det M\right|}{{\left|M_{22}\right|}^{2}}$$

where *M* is a 2 × 2 product matrix, *M*_22_ is the quantity of the second row, and the second column in this matrix $M=\prod_{l=0}^{N}M_{l}$ with transfer matrices *M*_l_ given by:

(13) $$M_{l}=\frac{1}{2}\left|\begin{array}{cc} \left(1+S_{l}\right)\exp\left[-i\left(k_{l+1}-k_{l}\right)x_{l}\right] & \left(1-S_{l}\right)\exp\left[-i\left(k_{l+1}+k_{l}\right)x_{l}\right]\\ \;\left(1-S_{l}\right)\exp\left[+i\left(k_{l+1}-k_{l}\right)x_{l}\right] & \left(1+S_{l}\right)\exp\left[+i\left(k_{l+1}-k_{l}\right)x_{l}\right] \end{array}\right|$$

In Equation 13, *S*_*l*_ = *m*_*l* + 1_*k*_*l*_/*m*_*l*_*k*_*l* + 1_, and the effective masses and momenta were discretized as *m*_*l*_ = m*[(*x*_*l* − 1_ + *x*_*l*_)/2] and *k*_*l*_ = *k*[(*x*_*l* − 1_ + *x*_*l*_)/2], respectively, *x*_l_ being the position of *l*th boundary. The Fermi-Dirac distribution was used in the tunneling current calculations, and the maximum of the longitudinal electron energy was set at 20 *k*_B_*T* above the conduction band.

## Results and discussion

The effective electron mass 0.5 *m*_0_ of SiO_2_, 0.26 *m*_0_ of silicon, 0.12 *m*_0_ of NC Ge [11] and the relative dielectric constant of the SiO_2_, Si, and Ge of 3.9, 11.9, and 16, respectively, have been used in calculations [12]. The published electron affinities of crystalline silicon, SiO_2_, and Ge are 4.05, 0.9, and 4.0 eV, respectively [13]. The thickness of the tunneling oxide layer and control oxide layer are 4 and 25 nm, respectively. *N*_A_ is 1 × 10^15^ cm^−3^, the temperature is 300 K, and the silicon substrate and gate are grounded in the following calculations.

**Figure 1:**
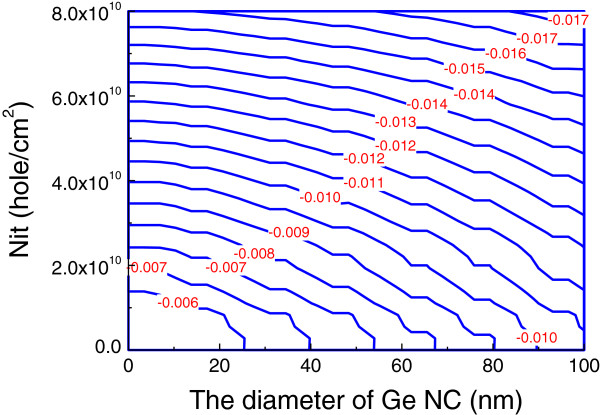
The contour of the voltage across the tunneling oxide layer.

The band banding becomes smaller with decreased stored electron in the NC Ge layer and leads to a decrease in the accumulation hole density [9]. A positive interface charge density leads to an increase in the electric field across the tunneling oxide layer, which is shown in Figure 1. It demonstrates that the electric field increases with the increase in the diameter of NC Ge at a stored charge in NC Ge layer of −1 × 10^12^ C. Similarly, we can prove that negative interface charge density will lead to a decrease in the electric field across the tunneling oxide layer. Figure 1 can be explained according to Equation 5 because *ψ*_s_ < 0, *Ε*_s_ < 0 and *Q*_it_ > 0 when *V*_g_ = 0.

**Figure 2:**
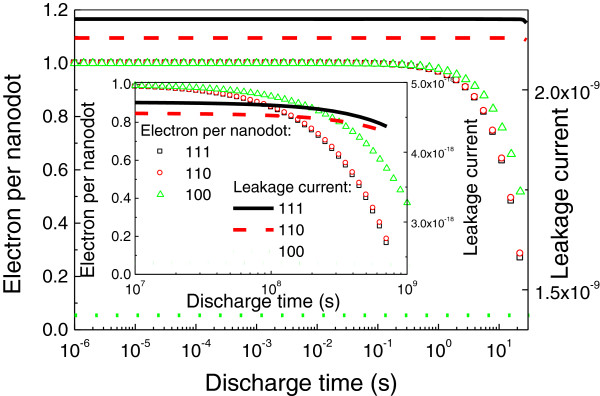
**Electron per NC and leakage current (A/cm**^**2**^**) as a function of time for different orientations.**

As we know, P_b_ defects at the Si and SiO_2_ interface for different silicon orientations have different characteristics [1]. Using the interface state energy distribution for the no H-passivation reported in [1], its effects on the discharging dynamics have been depicted in Figure 2. This figure clearly demonstrates that different silicon orientations have effects on the discharge dynamics when *d* = 8.4 nm and inset for *d* = 35 nm. A very small difference between those for Si(111) and Si(110) origins from the smaller difference between their leakage current (the largest relative difference is 3.3%) but increases with time. This is because at the initial stage, the quantity of the charge escaped from the NC Ge layer compared to the total quantity which is so small that the relative change cannot be observed from the figure.

The results for Si(100) can be easily explained because of the larger leakage current difference from those for Si(111) and Si(110). The leakage current exponentially increases due to a large increase in the *E*_c_ according to Equation 9 that leads to the leakage current exponentially increase. It implies that the ratio of the effects of interface charge on the leakage current to that of the *E*_c_ becomes smaller, and thus, the difference between those for different silicon orientations become smaller with the decrease in the diameter of NC. Whatever they have is the same trend for the different diameters.

**Figure 3:**
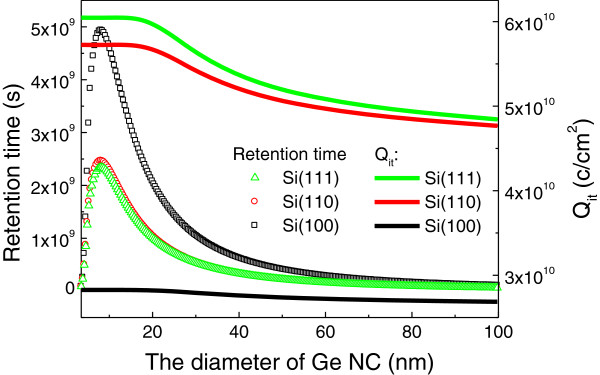
The retention time and initial interface charge density as a function of the diameter of NC Ge.

Figure 3 shows that the retention time firstly increase then decreases with the decrease in the diameter of NC when it is a few nanometers. The retention time is defined as 50% of the charges escaped from the NC Ge layer. As a comparison, the interface charge density for different silicon orientations and diameter is also depicted. It can be found that the Si(100)/SiO_2_ interface have the largest retention time due to the minimum leakage current. This figure illustrates that avoiding the size of NC Ge less than 4 nm can improve retention time when every NC is charged with one electron. Note that the average density of NC Ge is inversely proportional to the square of the thickness of NC Ge layer; it implies that smaller dimension of NC Ge layer stores more electrons for the case of per NC having one electron. Further, *E*_c_ changes slowly when the NC is tens of nanometers; whereas, it changes very fast when it is a few nanometers and leads a large reduction in the barrier height according to Equation 9 and linearly decreases with interface charge. Thus, the phenomenon of the retention time which firstly increases, then decreases with the decrease in the diameter, can be explained. The experimental data is that the average retention time is larger than 90 s when the average diameter of the nanocrystals is 8 nm with a standard deviation of 2.1 nm [14,15], whereas the retention time is smaller than 70 s when the average diameter of the nanocrystals is 5.67 nm with a standard deviation of 1.31 nm [16]. They qualitatively support the theoretical expectation.

## Conclusions

In conclusion, the effects of P_b_ defects at Si(100)/SiO_2_ interface for different silicon orientations on the discharging dynamics of NC Ge memory devices have been theoretically investigated. The results demonstrate that the Si(100)/SiO_2_ interface have the best discharge dynamics, and Si(110)/SiO_2_ and Si(111)/SiO_2_ interface are nearly same. It is also found that the retention time firstly increases, then decreases with the decrease in the diameter of NC when it is a few nanometers. The results also demonstrate that the effects of the interface traps on the discharge dynamics of NC Ge memory devices should be seriously taken into account. The experimental data reported in the literature [14,15] support the theoretical expectation.

## Competing interests

The author declares that he/she has no competing interests.

## Authors’ information

Ling-Feng Mao received the Ph.D degree in Microelectronics and Solid State Electronics from the Peking University, Beijing, People's Republic of China, in 2001. He is a professor in Soochow University. His research activities include modeling and characterization of quantum effects in MOSFETs, semiconductors and quantum devices and the fabrication and modeling of integrated optic devices.
